# Impact of the COVID-19 pandemic on anesthesia residency education

**DOI:** 10.36834/cmej.70457

**Published:** 2020-09-23

**Authors:** Jennifer M. O’Brien, Megan Deck, Una Goncin, Malone Chaya

**Affiliations:** 1 Department of Anesthesiology, Perioperative Medicine and Pain Management, College of Medicine, University of Saskatchewan, Saskatchewan, Canada

## Introduction

On March 11, 2020, the World Health Organization declared a global pandemic in response to the rapid spread of the novel coronavirus SARS-CoV-2.^1^ This declaration prompted accrediting colleges, public health authorities, and universities to release policies directly impacting specialty trainees.^2^^-^^4^

Resident trainees have expressed anticipatory loss over learning opportunities and qualifying exams, and a desire to continue their training.^5^ They have also expressed anxiety over their safety and that of their loved ones.^6^ Similarly, a qualitative study of internal medicine residents in Toronto during the SARS epidemic identified resident concerns regarding the negative impact on their educational experiences and patient care, personal safety, and emotional well-being.^7^

The clinical role of anesthesia residents during the COVID-19 pandemic has not been well described. As qualified physicians trained in airway management, some have argued anesthesia residents should be considered essential personnel,^8^ deployed in the event of human resource shortages.^2^^,^^3^ Conversely, others have argued that by deploying resident physicians in this environment, decision-makers are not adequately protecting the welfare of the lowest-paid doctors on the front lines given the uncertain supply of protective equipment.^9^

Given the diverse response of Canadian anesthesia residency programs,^10^^-^^12^ and the lack of a standardized framework for anesthesia residency training during a pandemic, we seek to answer the following questions:

- What are residents’ perceptions of the impact of the COVID-19 pandemic on their education?
- What are the strategies taken by different anesthesia programs to involve or not involve residents?
- What are residents’ attitudes towards these strategies?
- How many residents were exposed to patients with COVID-19 nationally, if working?

These results may inform the Royal College of Physicians and Surgeons, program directors, and health officials in optimizing anesthesia residency training during future pandemic conditions.

## Methods

We propose a mixed- methods study using a short questionnaire and a semi-structured interview with anesthesia residents from across Canada. We developed the survey according to established methodology.^13^ Through review of scholarly literature, news media, and informal conversations with colleagues, we deemed the following themes to be pertinent: personal safety, patient care, education, communication and leadership. We pre-tested the survey with three residents to improve relevance, clarity, and flow of questions.

Survey participants will be recruited through an invitation email with implied consent distributed by their residency program; respondents may indicate their desire to be contacted for a follow up interview. Interviewers will keep field notes for each interview, including impressions, reflections, and pragmatic notes. Interviews will be transcribed, and transcripts returned to participants to add, amend, or delete sections of their transcript as they choose. We encourage anesthesia program directors and residents to contact us to discuss opportunities for collaboration.

Survey responses will be reported in aggregate using descriptive statistics. Interview transcripts will be analyzed using thematic analysis,^14^^,^^15^ with the aim of exploring the lived experience of anesthesia residency education in the context of the COVID-19 pandemic. Thematic analysis allows us to examine the ways residents make meaning out of their experiences, and the ways in which these experiences are shaped by policy and context. Themes will be determined primarily based on their contribution to answering the research questions, rather than by their frequency across the dataset. We will create initial codes by highlighting key words and phrases and by connecting themes in the margin. Through an iterative process, the codes for recurring themes, thoughts, beliefs, experiences, and opinions will be reviewed and revised.

## Summary

This national survey of Canadian anesthesia residents will develop our understanding of medical education, safety, and perceptions towards training in the context of the COVID-19 pandemic. Our results may inform the Royal College of Physicians and Surgeons, program directors, and health officials in optimizing anesthesia residency training during future pandemic conditions.
